# An Evaluation of Different Partitioning Strategies for Bayesian Estimation of Species Divergence Times

**DOI:** 10.1093/sysbio/syx061

**Published:** 2017-07-04

**Authors:** Konstantinos Angelis, Sandra Álvarez-Carretero, Mario Dos Reis, Ziheng Yang

**Affiliations:** 1 Department of Genetics, Evolution and Environment, University College London, UK; 2 School of Biological and Chemical Sciences, Queen Mary University of London, London E1 4NS, UK

**Keywords:** Bayesian inference, genomic data, infinite-sites theory, molecular clock dating, partition analysis

## Abstract

The explosive growth of molecular sequence data has made it possible to estimate species divergence times under relaxed-clock models using genome-scale data sets with many gene loci. In order to improve both model realism and to best extract information about relative divergence times in the sequence data, it is important to account for the heterogeneity in the evolutionary process across genes or genomic regions. Partitioning is a commonly used approach to achieve those goals. We group sites that have similar evolutionary characteristics into the same partition and those with different characteristics into different partitions, and then use different models or different values of model parameters for different partitions to account for the among-partition heterogeneity. However, how to partition data in practical phylogenetic analysis, and in particular in relaxed-clock dating analysis, is more art than science. Here, we use computer simulation and real data analysis to study the impact of the partition scheme on divergence time estimation. The partition schemes had relatively minor effects on the accuracy of posterior time estimates when the prior assumptions were correct and the clock was not seriously violated, but showed large differences when the clock was seriously violated, when the fossil calibrations were in conflict or incorrect, or when the rate prior was mis-specified. Concatenation produced the widest posterior intervals with the least precision. Use of many partitions increased the precision, as predicted by the infinite-sites theory, but the posterior intervals might fail to include the true ages because of the conflicting fossil calibrations or mis-specified rate priors. We analyzed a data set of 78 plastid genes from 15 plant species with serious clock violation and showed that time estimates differed significantly among partition schemes, irrespective of the rate drift model used. Multiple and precise fossil calibrations reduced the differences among partition schemes and were important to improving the precision of divergence time estimates. While the use of many partitions is an important approach to reducing the uncertainty in posterior time estimates, we do not recommend its general use for the present, given the limitations of current models of rate drift for partitioned data and the challenges of interpreting the fossil evidence to construct accurate and informative calibrations.

It is well recognized that different parts of the genome are evolving at different rates with different patterns (such as different transition/transversion rate bias and different base composition bias) (Yang et al. 1995; Springer et al. 1999; Shapiro et al. 2006). Even the evolutionary histories (gene trees and divergence times) may differ among genes or genomic regions because of processes such as lateral gene transfer, gene duplication and loss, and deep coalescence due to ancestral polymorphism (Maddison 1997; Nichols 2001; Szollosi et al. 2014). With large molecular data sets typically analyzed in phylogenetic studies (Meusemann et al. 2010; dos Reis et al. 2012; Jarvis et al. 2014; Misof et al. 2014), there is an increasing need to accommodate the heterogeneity in evolutionary characteristics across sites or regions of the genome. For Bayesian divergence time estimation under relaxed-clock models, the heterogeneity among genomic regions in the relative substitution rates and in the patterns of substitution rate drift over branches of the phylogeny is expected to be particularly important.

There are mainly two kinds of models to deal with such among-site or among-region heterogeneity: mixture models and partition models. These correspond to “random-effects” and “fixed-effects” models in statistics, respectively. Take the modelling of substitution rate variation among sites in the same gene or sequence as an example. A mixture model assumes different classes of sites with different rates, but *a priori* we do not know which site is from which site class. This group of models includes the finite-mixture model (Yang et al. 1995), the infinite-mixture (Dirichlet-process) model (Lartillot and Philippe 2004; Huelsenbeck and Suchard 2007; Lartillot et al. 2009), and the continuous gamma model (Yang 1993; Mayrose et al. 2005). The parameters in the model include the probabilities and relative rates for the site classes in the finite-mixture model or the parameters in the gamma distribution or Dirichlet process. In a partition model, biological knowledge is used to group sites or genes into different partitions, with sites in the same partition sharing similar evolutionary characteristics while those in different partitions having different characteristics (Nylander et al. 2004; Brown and Lemmon 2007). One knows which sites are in which partition *a priori*. For example, the three codon positions have different rates and base compositions and may be treated as different partitions (Yang et al. 1995; Yang 1996; Shapiro et al. 2006). The two kinds of models are often combined, with the partitions accounting for large-scale differences in while the mixture model accommodates fine-scale variation among sites in the same partition. It is important to use biological knowledge to formulate partition models. For example, it is ill-advised to use an automatic algorithm to partition all sites in a super-alignment into different partitions, as this runs into a problem of data dredging and risks lumping similar observed sites (e.g., constant sites from all three codon positions) into spurious partitions.

What evolutionary characteristics are important and should be accounted for by the use of partitions should depend on the analysis. For divergence time estimation under relaxed-clock models, an important factor may be the rate differences among partitions and the different processes of rate drift over branches among partitions. In this article, we consider the species tree and divergence times to be fixed and shared by all partitions. Even in this simple case, choosing an appropriate partition scheme for divergence time estimation is challenging, partly because our knowledge of the process of sequence evolution (in particular, how evolutionary rate drifts over lineages and among genomic regions) is far from perfect and partly because fossil calibrations (which are critical in a dating analysis) are fraught with uncertainties and errors. The common practice has been to define partitions by genes or codon positions, or according to whether the sites are coding or noncoding, or whether they are from mitochondrial or nuclear genomes (Ho and Lanfear 2010; dos Reis et al. 2012; Jarvis et al. 2014). An automatic approach, known as PartitionFinder, starts from user-defined data subsets (also called data blocks) and iteratively merges the sets according to the Bayesian information criterion (BIC) (Lanfear et al. 2012, 2014). This is mostly designed for selecting partition models for phylogenetic tree reconstruction but is also used in divergence time estimation. Because the number of possible partition schemes is often too large (Lanfear et al. 2012), heuristic algorithms are used in the search for the best-fit scheme. ClockstaR is another automatic approach for “estimating” the best partition scheme for a given data set, especially suited to Bayesian divergence time estimation (Duchêne et al. 2014). This uses the maximum likelihood estimates of branch lengths on the fixed unrooted species tree topology for each data block and calculates a distance metric between data blocks to measure whether the branch lengths are proportional between them. A clustering algorithm is then used to partition the data blocks and to assign data blocks to partitions. Duchêne and Ho (2014) used simulation to demonstrate the utility of ClockstaR for partitioning data in molecular clock dating analyses.

The choice of partition schemes may affect downstream phylogenetic analyses. Several studies have examined the effect of data partitioning on the inference of tree topology (Strugnell et al. 2005; Leavitt et al. 2013), finding that under partitioning may lead to highly supported but incorrect nodes on the estimated tree (Kainer and Lanfear 2015). However, there has been no systematic effort to explore the effect of partitioning on the estimation of species divergence times under the clock or relaxed-clock models. Poux et al. (2008) and Voloch and Schrago (2012) found that different partition schemes produced similar posterior divergence time estimates. However those studies used closely related species so that the molecular clock holds approximately, and the conclusions may not apply in general to relaxed clock dating with serious clock violation. According to the infinite-sites theory, increasing the number of partitions is essential to improving the precision of posterior time estimation in relaxed-clock dating if the fossil calibrations are fixed (Rannala and Yang 2007; Zhu et al. 2015). We thus expect the choice of data partition schemes to have a major impact on the accuracy and precision of divergence time estimation in a relaxed-clock dating analysis.

Here, we explore the performance of several commonly used partition schemes on Bayesian estimation of species divergence times using simulated data of multiple protein-coding gene sequences, including concatenation, partitioning by codon position, and by gene. We simulate sequence alignments from a nine-species phylogeny with known node ages and analyze them to estimate the divergence times using six partition schemes. We study two cases of clock violation (slight and severe clock violations) and examine the impact of various factors (such as the prior on the rate-drift process, the number and quality of fossil calibrations) on divergence time estimation, when the sequence data are partitioned using different strategies.

## Methods

### Design of the Simulation Experiment

**Figure 1.**
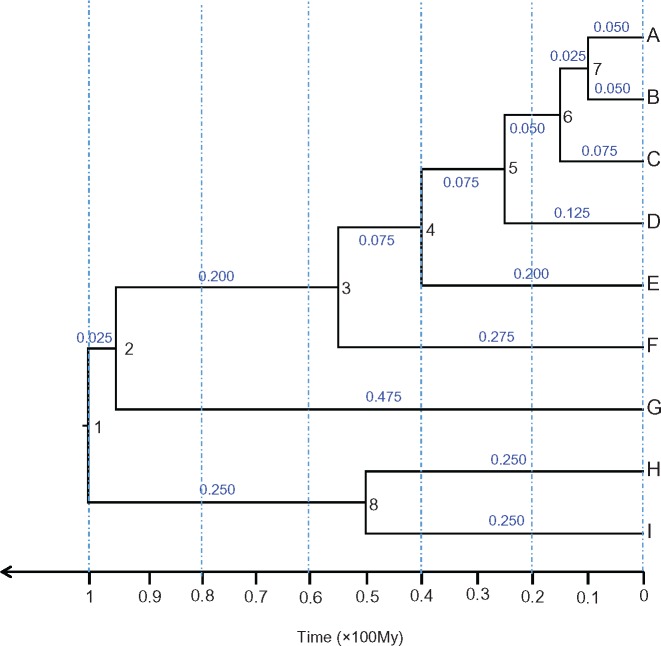
Species tree with node ages used to simulate gene alignments. Internal nodes are numbered 1 to 8, and their ages are indicated by the time axis. Branch lengths are shown next to the branches, calculated assuming a substitution rate of $5 \times 10^{\mathrm{-9}}$ substitutions per site per year throughout the tree.

We use the nine-species phylogeny of Figure 1 to simulate 50 alignments of protein-coding genes. The divergence times are fixed at $t_{\mathrm{1}} = 1$ (for the root), $t_{\mathrm{2}} = 0.95$, $t_{\mathrm{3}} = 0.55$, $t_{\mathrm{4}} = 0.40$, $t_{\mathrm{5}} = 0.25$, $t_{\mathrm{6}} = 0.15$, $t_{\mathrm{7}} = 0.10$, and $t_{\mathrm{8}} = 0.50$. One time unit is 100 myr so that the root age is 100 myr. We chose to use a fixed tree and fixed divergence times so that the simulation results are easily interpretable and we do not expect the tree topology to have major effects on our results (relative to other factors that we consider such as the number and quality of the fossil calibrations and the prior model of evolutionary rate drift). We simulate random rates for branches using an independent-rates (IR) model but allow variable rates among genes. The branch rate here should be considered an average over the branch as the sequence data is not informative about rate variation within a branch. We set the overall rate across lineages for the $g$th gene to be a random variable from the gamma distribution, $\mu_{g}$ ~ $G$$10,\,\,10/{{\rm{\mu }}_0})$, with mean $\mu_{\mathrm{0}} = 0.5$ (0.5 substitutions per site per time unit or $5 \times 10^{\mathrm{-9}}$ substitutions per site per year) and the 95% interval (0.24, 0.85). The log-rates for the branches of the $g$th gene are generated as independent random variables from the normal distribution, log $\mu_{gb}$ ~ $N$(log $\mu_{g} - \sigma ^{\mathrm{2}}$/2, $\sigma^{\mathrm{2}})$, for $b = 1$, …, 16. Note that this log-normal distribution has mean E($\mu_{gb}) = \mu_{g}$ and variance V($\mu_{gb}) = \left[ {\exp \{\sigma^{2}\}-1} \right]\mu_{g}^{2} $, with coefficient of variation (standard deviation/mean) to be $\sqrt {\exp \{\sigma^{2}\}-1} $, so that $\sigma^{\mathrm{2}}$ is a scale-free measure of the violation of the clock, independent of the time unit (Brown and Yang 2011). We multiply $\mu_{gb}$ with the time duration of the $b$th branch to calculate the branch length on the $g$th gene tree. In this way, we construct 50 gene trees with branch lengths. We use two values for the variance parameter of the rate-drift process: $\sigma^{\mathrm{2}} = 0.01$ and 0.25, corresponding to slight and serious clock violations respectively. A previous simulation found that at $\sigma^{\mathrm{2}} = 0.01$, the likelihood ratio test rarely rejected the clock and the Bayesian credibility intervals under the strict clock model included the true ages, while at $\sigma ^{\mathrm{2}} = 0.25$, the likelihood ratio test almost always rejected the clock and the posterior intervals under the strict clock rarely included the true ages (Brown and Yang 2011, Figs. 1 and 3). In either case, the 50 genes may have different overall rates, but all genes have the same extent of among-branches rate variation (the same $\sigma ^{\mathrm{2}})$. Note that according to our simulation design, all genes share the same tree topology and divergence times, but they have independent overall rates, and given the overall rates, the branch rates vary independently among genes. Simple R code is written to sample the branch rates and to generate the gene trees.

**Figure 3.**
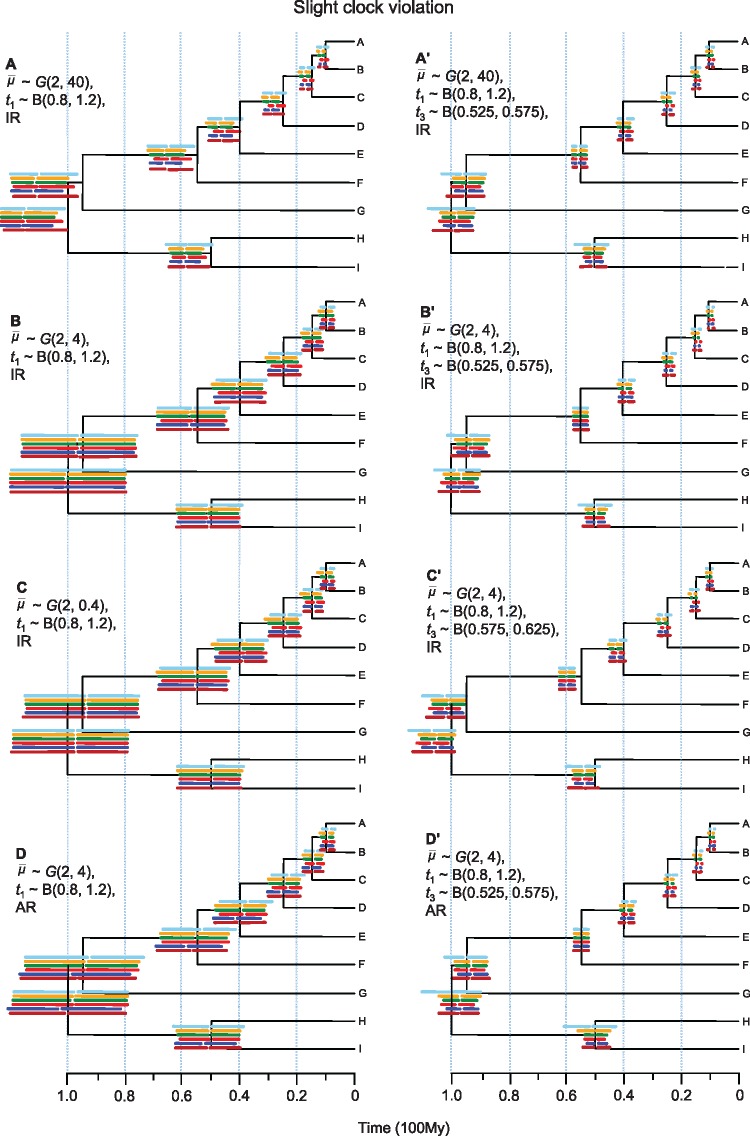
Posterior divergence time estimates from simulated data when the clock is slightly violated. See caption of Figure 2.

The generated gene trees have branch lengths measured in substitutions per site. We multiply all branch lengths by 3 as there are ~ 3 nucleotide sites in each codon. Gene sequence alignments are then simulated on the gene trees under the M3 (discrete) model of codon evolution (Yang et al. 2000) using the program EvolverNSsites from PAML v4.8 (Yang 2007). This model allows for three classes of codons with different nonsynonymous to synonymous rate ratios: $\omega _{\mathrm{0}} = $0.01, $\omega _{\mathrm{1}} = $0.5, and $\omega _{\mathrm{2}} = $0.9. We simulate 25 conserved genes with probabilities $p_{\mathrm{0}} = 0.8$, $p_{\mathrm{1}} = 0.19$, and $p_{\mathrm{2}} = 0.01$ for the three site classes, with the average $\omega $ to be 0.112; and 25 less-conserved genes with probabilities $p_{\mathrm{0}}$$=$ 0.5, $p_{\mathrm{1}} = 0.3$, and $p_{\mathrm{2}} = 0.2$, with the average $\omega $ to be 0.335. The sequence length of each gene is $n = 500$ codons, the transition/transversion rate ratio is $\kappa = 2$ and the codon frequencies are assumed to be equal. The number of replicates is 100. In total, $2 \times 100$ data sets were simulated, each consisting of 50 genes, with 100 data sets for $\sigma^{\mathrm{2}} = 0.01$ and 100 for $\sigma^{\mathrm{2}} = 0.25$.

### Estimation of Divergence Times from the Simulated Gene Alignments

We analyzed the simulated gene alignments with the program MCMCTREE v4.8 (Yang 2007) to estimate the species divergence times. We evaluated the following partition schemes, which are commonly used in phylogenomic studies:

1) We concatenated all genes into a single “supergene” (concatenation, C).
2) We concatenated the first and second codon positions from all genes into one partition and the third codon positions from all genes into another (codon position, CP).
3) Ministry of Agriculture and Rural DevelopmentMinistry of Agriculture and Rural DevelopmentWe used the program PartitionFinder v1.1.1 (Lanfear et al. 2012, 2014), with codon positions $1 + 2$ and 3 of each gene treated as two separate data blocks (PartitionFinder, PF). The program explores different partitioning strategies using the BIC. The number of inferred partitions ranges from 1 to 100.
4) We analyzed the data as 50 partitions with each partition to be a gene (gene, G).
5) We treat the first and second codon position of each gene as one partition and the third codon positions as another, creating in total $2 \times 50 = 100$ partitions (gene and codon position, GCP).
6) We used ClockstaR v2.0.1 (Duchêne et al. 2014) as another automatic method for determining the partition scheme (ClockstaR, CS). As with PartitionFinder, we use 100 data blocks per replicate data set. Branch lengths on the fixed unrooted tree were estimated using BASEML (Yang 2007) for each data block. These were then used as input for ClockstaR to calculate a distance metric between data blocks and the resulting distance matrix was used to cluster data blocks into partitions.

The PartitionFinder analysis may merge (concatenate) different data blocks into one partition, but will never separate sites in the same data block into different partitions. The program estimates the best-fitting partition scheme and the best-fitting substitution model for each partition from a user-specified set of models based on an information criterion. The tree topology is either provided by the user or estimated from the data. We used the 100 data blocks defined in the GCP scheme as the starting point and the fixed tree of Figure 1. We did not search for the best-fitting substitution model for each partition but used HKY85$+\Gamma_{\mathrm{4}}$ throughout. We note that automatic model selection (e.g., Posada and Crandall 1998) often leads to parameter-rich pathological models, such as the “I$+\Gamma $” model, and that furthermore the use of different substitution models for the same data blocks in different partition schemes may compromise the comparison of partition schemes. Note that with different parameter values for partitions, the HKY85$+\Gamma_{\mathrm{4}}$ model is capable of accommodating the heterogeneity (among partitions) in the substitution rate, base compositions, transition/transversion rate ratio, and the extent of among-site rate variation.

We used the *greedy* heuristic algorithm with the BIC score to search for the best scheme since it was found to perform better than other algorithms (i.e., *rcluster* and *hcluster*), although it requires more computation (Lanfear et al. 2014). We used the linked option for branch length estimation by which one set of branch lengths is estimated and a scaling parameter is used to adjust the branch lengths for each partition.

The ClockstaR program runs in three steps: (i) estimating the best-fitting substitution model using the BIC score and the branch lengths on the fixed unrooted tree using maximum likelihood for each data block, (ii) estimating the distance for each pair of data blocks, and (iii) using cluster analysis to find the optimal number of partitions and to assign data blocks to partitions. We used BASEML to estimate the branch lengths for each of the 100 data blocks under HKY85$+\Gamma_{\mathrm{4}}$. As in the case of PartitionFinder, we used the same model for all data blocks. The function trees.bsd was then used to estimate the sBDS$_{\mathrm{min}}$ distance metric between partitions and the partitions.object function was used for the cluster analysis. This calculates the Gap statistic (Tibshirani et al. 2001) for each number of partitions ($k)$, using 500 bootstrap replicates (Duchêne et al. 2014). The lowest $k$ that triggers a peak in the Gap statistic is the optimal. Data blocks were assigned to partitions by applying the Partitioning Along Medoids algorithm (Kaufman and Rousseeuw 2009).

We set the time unit in MCMCTREE to 100 myr and applied three calibration strategies. (i) We assigned the calibration 0.8 < $ t_{\mathrm{1}}$ < 1.2 on the root age, represented by the calibration density $t_{\mathrm{1}}$ ~ B(0.8, 1.2). Here “B” stands for a pair of bounds, represented by a soft uniform distribution, with left and right tail probabilities 2.5% that the root age is outside the bounds (Yang and Rannala 2006, Fig. 2c). This mimics a soft-bound calibration on the root between 80 Ma and 120 Ma based on the fossil record. (ii) We applied the same constraint on the root age 0.8 < $t_{\mathrm{1}}$ < 1.2, and in addition the constraint 0.525 < $t_{\mathrm{3}}$ < 0.575 on node 3, mimicking a weak calibration on the root and an informative calibration on node 3. (iii) We used the same constraint on the root age and a conflicting constraint $t_{\mathrm{3}}$ ~ B(0.575, 0.625) on node 3. Since the true age ($t_{\mathrm{3}} = 0.55$) is outside those bounds, this mimics an incorrect calibration on node 3. The prior for the ages of the uncalibrated internal nodes was specified using a birth–death sampling process with birth and death rates $\lambda = \mu = 1$ and sampling fraction $\rho = 0$, which represents a uniform kernel (Yang and Rannala 2006). We ran the MCMCTREE program without data and confirmed that the marginal time priors on the calibrated nodes closely matched the user-specified densities.

**Figure 2.**
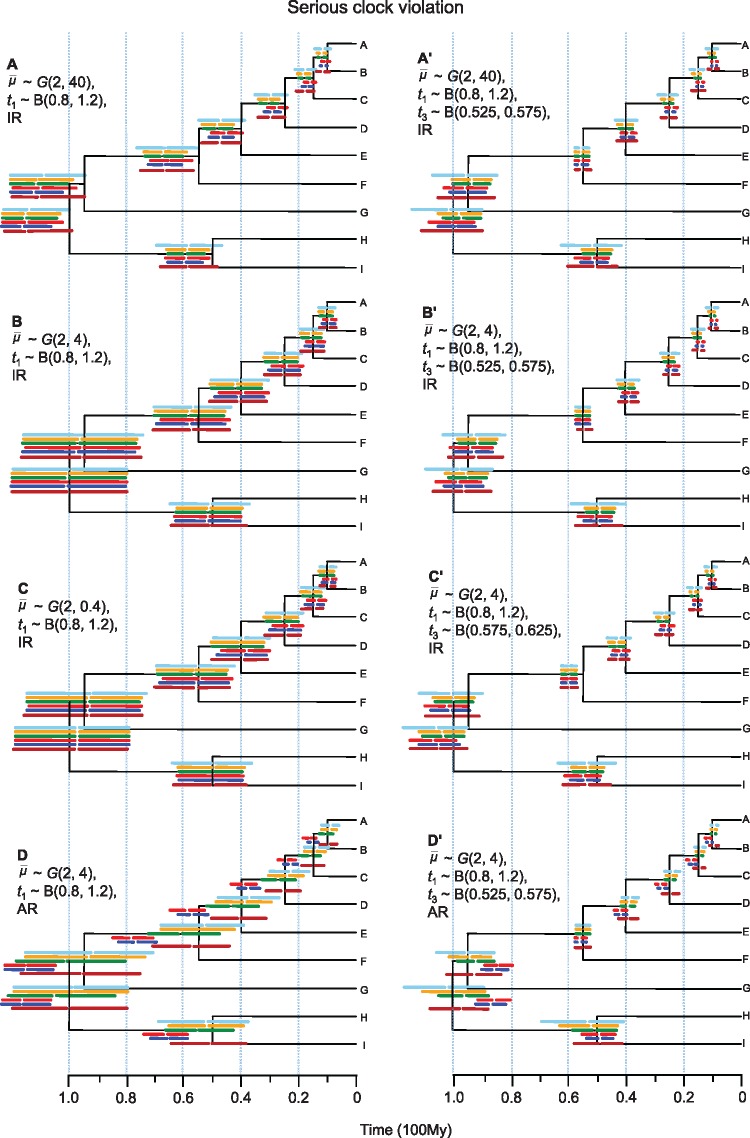
Posterior divergence time estimates from simulated data for different combinations of rate prior, calibration strategy and rate-drift model, when the clock is seriously violated. The tree shown is the true timetree. Horizontal bars represent the 95% highest posterior density (HPD) intervals for the six partition schemes. These are (from top to bottom): (i) concatenation (C), 1 single partition; (ii) codon positions (CP, 2 partitions); (iii) PartitionFinder (PF, variable partitions); (iv) gene (G, 50 partitions); (v) both gene and codon positions (GCP, 100 partitions); and (vi) ClockstaR (CS, variable partitions). The gap within the bar represents the posterior mean. The time estimates and their intervals are averages over the 100 replicates. IR $=$ independent-rates model, AR $=$ autocorrelated-rates model.

We used the relaxed-clock model implemented in MCMCTREE to analyze the data (Rannala and Yang 2007). The IR model assumes that given the overall rate for the locus, the rates for branches on the tree are independent log-normal variables. The overall locus rates are assigned the gamma-Dirichlet prior (dos Reis et al. 2014): a gamma prior is assigned to the average rate over all loci ($\bar{\mu}$) and the locus rates are then assigned by partitioning the total rate according to the uniform Dirichlet distribution. We used $\bar{\mu} \sim G$(2, 4) with mean 0.5, meaning $5 \times 10^{-9}$ substitutions per site per year with prior 95% interval (0.06, 14.0). The mean of this prior matches the overall substitution rate ($\mu _{0} = 0.5$) of all genes in the simulation but the shape parameter (2) means that the prior is fairly diffuse. In addition we used two “incorrect” rate priors, to assess the performance of the partition schemes under incorrect rate priors: (i) a slow rate, $\bar{\mu} \sim G$(2, 40) and (ii) a fast rate $\bar{\mu} \sim G$(2, 0.4). We also used the autocorrelated-rates model (AR) in which the branch rates evolve from the rate at the root according to the geometric Brownian motion (Thorne et al. 1998; Thorne and Kishino 2002; Rannala and Yang 2007). A gamma prior, $\bar{\sigma}^2 \sim G$(2, 20), was assigned to the average rate drift parameter ($\sigma^{\mathrm{2}})$ among loci with the locus-specific parameters to be defined from the Dirichlet distribution. The topology of Figure 1 was used along with the HKY85$+\Gamma_{\mathrm{4}}$ model of nucleotide substitution. The approximate likelihood method was used for computational efficiency (dos Reis and Yang 2011). The test carried out by dos Reis and Yang (2011) (see also Inoue et al. 2010) suggests that the approximation is acceptable with $\geqslant $100 sites while here each partition has at least 500 sites (if the partition has only one data block of codon position 3).

Markov chain Monte Carlo (MCMC) was run with a burn-in of 10$^{\mathrm{6}}$ steps, sampling every 500 steps to collect 10$^{\mathrm{4}}$ samples from the posterior. For the partitioning strategy GCP, posterior samples were collected every 250 steps to save computational time. Convergence was evaluated for only the first replicate for each combination of rate prior, calibration strategy, rate-drift model, and partition scheme by running two independent MCMC runs with different starting values. For each replicate we estimated the posterior means and the 95% high posterior density (HPD) intervals of divergence times. Those are averaged over the 100 replicates to assess the performance of different partition schemes.

### The Performance of Different Partitioning Strategies

We evaluate different partition strategies by using four measures of accuracy and precision of posterior time estimates. Each measure is calculated for every node in the species tree (Fig. 1) as an average over the replicate data sets, and then averaged over all nodes.

(i) Average relative error. We calculate the relative error of the time estimate for node $i$ in the $j$th replicate $d_{ij} = \left| {\tfrac{\tilde{{t}}_{ij} \sim t_{i} }{t_{i} }} \right|$, where $t_{i}$ is the true age of node $i$ and $\tilde{t}_{ij}$ is its estimate (the posterior mean), with $j = 1,..., 100, i = 1,..., s-1$, where $s$ is the number of species. This may be considered a measure of accuracy for the point estimate.
(ii) Relative HPD width. We calculate the relative HPD interval width of the time estimate for node $i$ in replicate $j$ as *sw*$_{ij} = w_{ij}/t_{i}$, where $w_{ij}$ is the 95% HPD interval for node age $t_{i}$ in replicate $j$. This is a measure of precision.
(iii) Mean square error (MSE). The square root of MSE of the time estimate of node $i$ in replicate $j$ is $\sqrt {\mbox{MSE}_{ij} } =\sqrt {V(\tilde{{t}}_{ij} )+(\tilde{{t}}_{ij} -t_{i} )^{2}} $, where $V(\tilde{{t}}_{ij} )\approx [{w_{ij} } /{(2\times 1.96)}]^{2}$. This is a measure of both accuracy and precision of the time estimates.
(iv) Coverage probability. For each node $i$, coverage ($P_{i})$ is calculated as the percentage of replicates in which the 95% HPD interval contains the true age $t_{i}$.

### Divergence Times of Plants

We estimated the divergence times of 15 plant species using the 6 partition schemes considered in the simulation. The molecular data are from Ruhfel et al. (2014) and consist of 78 plastid gene alignments (58,347 sites in total). Note that here the G scheme involves 78 partitions (one for each gene) while GCP involves 156 ($=78 \times 2$; for codon positions 1 and 2 vs. 3 for every gene).

We used PartitionFinder and ClockstaR with the same settings as in the simulation analysis, except that the GTR$+\Gamma_{\mathrm{4}}$ model was used to estimate branch lengths by maximum likelihood for each data block. We used three priors for the average rate, $\bar{\mu}\sim G$(1, 100), $\bar{\mu}\sim G$(1, 10), and $\bar{\mu}\sim G$(1, 1) (Magallon et al. 2013), with the prior mean rate to be 10$^{\mathrm{-10}}$, 10$^{\mathrm{-9}}$, and 10$^{\mathrm{-8}}$ substitutions per site per year. The time prior was constructed from the calibrations together with the birth–death sampling process, with a uniform kernel ($\lambda = \mu = 1$, and $\rho = 0$). For the rate-drift parameter, we used the prior $\bar{\sigma}^2\sim G$(1, 10). The GTR$+\Gamma _{\mathrm{4}}$ substitution model was used in all partitions and approximate likelihood calculation was used to save computational time (dos Reis and Yang 2011). We used both the IR and the AR models for the among-branches rate variation.

All MCMC analyses were run with the same settings as in the simulation. Two MCMC runs were used for each analysis to confirm convergence.

## Results

The six partition schemes we evaluated by simulation were: (i) concatenation (C, 1 partition); (ii) codon positions (CP, 2 partitions); (iii) PartitionFinder (PF, variable number of partitions); (iv) gene (G, 50 partitions); (v) gene and codon positions (GCP, 100 partitions), and (vi) ClockstaR (CS, variable number of partitions). The number of partitions determined by PartitionFinder varied from 9 to 17 among the replicate data sets with serious clock violation ($\sigma^{\mathrm{2}}$$=$ 0.25) and from 9 to 16 with mild clock violation ($\sigma^{\mathrm{2}} = 0.01$). ClockstaR showed much larger variations among data sets, with the number ranging from 1 to 96 for the nonclock-like data and from 1 to 50 for clock-like data. There was little correlation between the two methods (Supplementary Fig. S1 available on Dryad at http://dx.doi.org/10.5061/dryad.d7839). We note that if the molecular clock holds, ClockstaR should ideally infer a single partition and be equivalent to concatenation, but surprisingly the method inferred a single partition for many data sets simulated with serious clock variation as well. Note that we generated the data under a codon-based substitution model so that there does not exist a “true” partition scheme.

### Results from Simulations When the Clock is Seriously Violated

The molecular clock is seriously violated in data sets simulated using $\sigma^{\mathrm{2}} = 0.25$. We evaluated different partition schemes using four performance measures: relative error, relative HPD width, MSE, and coverage probability. The relative error, relative HPD width, and MSE averaged over the 100 replicates and over all internal nodes are shown in Table 1, while the results for two representative nodes (node 1, the root, with a fossil calibration; and node 4 without calibration) are shown in Table 2. Corresponding results on coverage are in Tables 3 and 4.

**Table 1.** Performance measures for different partitioning strategies when the clock is seriously violated

			Relative error						HPD width/age						$\sqrt{\rm MSE}$					
Model	$\bar{\mu}$	Calibration	C (1P)	CP (2P)	G (50P)	GCP (100P)	PF (V)	CS (V)	C (1P)	CP (2P)	G (50P)	GCP (100P)	PF (V)	CS (V)	C (1P)	CP (2P)	G (50P)	GCP (100P)	PF (V)	CS (V)
IR	***G*(2, 40)**	$t_{\mathrm{1}}\sim $ B(0.8, 1.2)	0.177	0.204	**0.138**	0.177	0.204	0.186	0.40	0.27	0.27	**0.22**	0.25	0.35	0.092	0.097	**0.082**	0.094	0.098	0.094
	$G$(2, 4)	$t_{\mathrm{1}}\sim $ B(0.8, 1.2)	**0.028**	0.046	0.039	0.039	0.048	0.036	0.50	0.45	**0.43**	**0.43**	0.44	0.48	0.060	0.058	**0.056**	**0.056**	0.058	0.060
	***G*(2, 0.4)**	$t_{\mathrm{1}}\sim $ B(0.8, 1.2)	**0.032**	0.036	0.052	0.037	**0.032**	**0.032**	0.50	0.45	0.43	**0.42**	0.44	0.48	0.060	0.057	0.057	**0.055**	**0.055**	0.059
	***G*(2, 40)**	$t_{\mathrm{1}}\sim $ B(0.8, 1.2)	0.030	0.032	0.040	0.032	0.030	**0.029**	0.27	0.16	0.16	**0.14**	**0.14**	0.23	0.034	0.024	0.026	**0.022**	0.023	0.030
		$t_{\mathrm{3}}\sim $ B(0.525, 0.575)																		
	$G$(2, 4)	$t_{\mathrm{1}}\sim $ B(0.8, 1.2)	**0.028**	0.030	0.046	0.040	0.032	0.029	0.26	0.16	0.17	**0.14**	0.15	0.22	0.033	0.026	0.027	**0.025**	0.026	0.030
		$t_{\mathrm{3}}\sim $ B(0.525, 0.575)																		
	***G*(2, 0.4)**	$t_{\mathrm{1}}\sim $ B(0.8, 1.2)	**0.029**	0.040	0.048	0.041	0.033	0.031	0.26	0.18	0.17	**0.14**	0.15	0.22	0.033	0.029	0.027	**0.026**	**0.026**	0.031
		$t_{\mathrm{3}}\sim $ B(0.525, 0.575)																		
	$G$(2, 4)	$t_{\mathrm{1}}\sim $ B(0.8, 1.2)	0.072	0.080	0.063	**0.062**	0.072	0.071	0.26	0.17	0.17	**0.15**	**0.15**	0.23	0.047	0.040	0.043	**0.038**	**0.038**	0.044
		***t*** $_{\mathrm{\mathbf{3}}}\sim $ **B(0.575, 0.625)**																		
**AR**	$G$(2, 4)	$t_{\mathrm{1}}\sim $ B(0.8, 1.2)	0.060	0.040	0.435	0.388	0.091	**0.036**	0.53	0.47	0.31	**0.26**	0.45	0.48	0.067	**0.059**	0.161	0.150	0.069	**0.059**
	$G$(2, 4)	$t_{\mathrm{1}}\sim $ B(0.8, 1.2)	0.044	0.036	0.101	0.084	0.034	**0.029**	0.25	0.19	0.15	**0.13**	0.17	0.22	0.037	0.030	0.051	0.045	**0.027**	0.030
		$t_{\mathrm{3}}\sim $ B(0.525, 0.575)																		

*Notes:* The performance measures are averages over the 100 replicates and over the 8 internal nodes on the tree. The partitioning strategies are C: concatenation (1 partition); CP: codon position (2P); PF: PartitionFinder (Variable); G: gene (50P); GCP: gene and codon position (100P); and CS: ClockstaR (Variable). Incorrect rate prior and calibrations are highlighted in bold. Cells in bold indicate the preferred partitioning strategy according to the respective measure.

With a single calibration $0.8 < t_{1} < 1.2$ and under the rate prior $\bar{\mu}\sim G$(2, 4) and the IR model, time estimates were close to the true values for all partition schemes (Fig. 2B). The relative error of the point estimates averaged over all nodes and replicates were 0.028, 0.046, 0.039, 0.039, 0.048, and 0.036 for partition schemes C (concatenation), CP (codon position), G (gene), GCP (gene and codon position), PF (PartitionFinder), and CS (ClockstaR), respectively, with scheme C being the most accurate (Table 1). The differences in time estimates among the partition schemes were small. The true ages were well within the HPD time intervals for all partition schemes (Table 1). Concatenation produced wider HPD intervals, with lower precision than all other schemes. According to the root MSE, which is a combined measure of both precision and accuracy, the G and GCP schemes performed the best (Table 1). We also note that some nodes on the tree were dated better than other nodes: the age of the root was estimated more accurately than those of other nodes for all partition schemes, apparently because the root was the only node with a calibration.

When we added another good fossil calibration on node 3, 0.525 < $t_{\mathrm{3}}$ < 0.575 (the true age is $t_{\mathrm{3}} = 0.55$), time estimates became more precise for all nodes and partition schemes (compare Fig. 2B’ with 2B). For example, the relative HPD width over all nodes decreased from 0.50 to 0.26 for the C scheme and from 0.43 to 0.17 for the G scheme (Table 1). Accuracy was either the same or improved for the partition schemes C, CP, PF, and CS but was slightly worse for the highly partitioned schemes G and GCP (Table 1). The age of node 3 was accurately and precisely estimated for all partition schemes owing to the informative calibration on it, whereas the age of the root was not accurately estimated in some partition schemes. For example, the relative error for the root age increased from 0.002 to 0.029 for the C scheme and from 0.003 to 0.030 for the G scheme after the inclusion of the additional calibration on node 3 (Table 2). All partition schemes except schemes C and CS had similar root MSE (i.e., 0.026, 0.027, 0.025, and 0.026 for schemes CP, G, GCP, and PF, respectively; Table 1) but the highly partitioned schemes G and GCP had smaller coverage probabilities (Table 3), with higher precision but lower accuracy (Table 1).

**Table 2.** Performance measures for nodes 1 (root) and 4 for different partitioning strategies when the clock is seriously violated

			Relative error						HPD width/age						$\sqrt{\rm MSE}$					
	$\bar{\mu}$	Calibration	C (1P)	CP (2P)	G (50P)	GCP (100P)	PF (V)	CS (V)	C (1P)	CP (2P)	G (50P)	GCP (100P)	PF (V)	CS (V)	C (1P)	CP (2P)	G (50P)	GCP (100P)	PF (V)	CS (V)
IR	***G*(2, 40)**	$t_{\mathrm{1}}\sim $ B(0.8, 1.2)	**0.141**	0.154	0.151	0.166	0.159	0.148	0.23	0.20	0.21	**0.17**	0.19	0.23	**0.153**	0.162	0.160	0.172	0.166	0.159
			0.183	0.216	**0.136**	0.188	0.218	0.195	0.40	0.27	0.27	**0.22**	0.25	0.35	**0.084**	0.091	0.061	0.078	0.091	0.086
	$G$(2, 4)	$t_{\mathrm{1}}\sim $ B(0.8, 1.2)	**0.002**	0.005	0.003	0.015	0.010	0.008	0.40	0.40	0.40	0.40	0.40	0.40	0.102	0.102	0.102	0.102	0.102	0.102
			**0.030**	0.054	0.033	0.038	0.056	0.040	0.50	0.45	**0.43**	**0.43**	0.44	0.48	0.053	0.052	**0.046**	0.047	0.052	0.053
	***G*(2, 0.4)**	$t_{\mathrm{1}}\sim $ B(0.8, 1.2)	0.027	0.026	0.027	**0.025**	0.026	0.026	0.40	0.40	0.40	0.40	0.40	0.40	0.105	0.105	0.105	0.105	0.105	0.105
			0.032	0.034	0.051	0.033	0.032	**0.031**	0.49	0.45	**0.42**	**0.42**	0.44	0.48	0.053	0.049	0.049	**0.046**	0.047	0.051
	***G*(2, 40)**	$t_{\mathrm{1}}\sim $ B(0.8, 1.2)	**0.027**	0.031	0.028	0.028	0.033	0.028	0.24	0.15	0.15	**0.13**	**0.13**	0.21	0.070	0.051	0.050	**0.045**	0.049	0.062
		$t_{\mathrm{3}}\sim $ B(0.525, 0.575)	**0.016**	0.020	0.026	0.019	0.018	0.017	0.18	0.12	0.13	**0.11**	0.12	0.16	0.020	0.016	0.018	**0.014**	0.015	0.018
	$G$(2, 4)	$t_{\mathrm{1}}\sim $ B(0.8, 1.2)	**0.029**	0.046	0.030	0.039	0.049	0.036	0.23	0.15	0.16	**0.13**	0.14	0.20	0.068	0.063	**0.053**	0.055	0.063	0.066
		$t_{\mathrm{3}}\sim $ B(0.525, 0.575)	0.026	**0.017**	0.040	0.029	0.019	0.024	0.19	0.13	0.14	**0.12**	**0.12**	0.17	0.022	0.016	0.022	0.018	**0.015**	0.020
	***G*(2, 0.4)**	$t_{\mathrm{1}}\sim $ B(0.8, 1.2)	**0.031**	0.048	**0.031**	0.041	0.051	0.038	0.23	0.15	0.16	**0.13**	0.14	0.20	0.069	0.065	**0.053**	0.057	0.065	0.067
		$t_{\mathrm{3}}\sim $ B(0.525, 0.575)	0.027	**0.020**	0.042	0.031	**0.020**	0.026	0.19	0.14	0.14	**0.12**	**0.12**	0.17	0.023	0.017	0.023	0.018	**0.016**	0.020
	$G$(2, 4)	$t_{\mathrm{1}}\sim $ B(0.8, 1.2)	0.063	0.045	0.073	0.054	**0.041**	0.055	0.22	0.15	0.16	**0.14**	**0.14**	0.20	0.087	0.063	0.085	0.068	**0.057**	0.077
		***t*** $_{\mathrm{\mathbf{3}}} \sim$ ** B(0.575, 0.625)**	0.067	0.086	**0.047**	0.062	0.079	0.070	0.20	0.14	0.14	**0.13**	**0.13**	0.17	0.034	0.037	**0.024**	0.028	0.035	0.034
**AR**	$G$(2, 4)	$t_{\mathrm{1}}\sim $ B(0.8, 1.2)	0.013	0.012	0.166	0.168	0.055	**0.007**	0.40	0.40	**0.17**	**0.17**	0.37	0.40	0.103	**0.102**	0.171	0.173	0.112	**0.102**
			0.071	0.041	0.478	0.433	0.096	**0.040**	0.53	0.47	0.30	**0.26**	0.45	0.48	0.063	**0.052**	0.194	0.175	0.062	**0.052**
	$G$(2, 4)	$t_{\mathrm{1}}\sim $ B(0.8, 1.2)	0.030	**0.029**	0.141	0.129	0.042	0.036	0.28	0.22	0.11	**0.10**	0.17	0.20	0.079	**0.065**	0.144	0.132	**0.065**	0.066
		$t_{\mathrm{3}}\sim $ B(0.525, 0.575)	0.039	0.027	0.054	0.046	**0.020**	0.024	0.15	0.12	0.11	**0.10**	0.12	0.17	0.022	0.017	0.025	0.022	**0.015**	0.020

*Notes:*The performance measures for each node are averages over the 100 replicates. The first row in each cell refers to node 1 (the root) and the second to node 4. See caption of Table 1 for more details.

**Table 3.** Average coverage for different partitioning strategies when the clock is seriously violated

Model	$\bar{\mu}$	Calibration	C (1P)	CP (2P)	G (50P)	GCP (100P)	PF (V)	CS (V)
IR	***G*(2, 40)**	$t_{\mathrm{1}}\sim $ B(0.8, 1.2)	**72**	7	46	11	5	56
	$G$(2, 4)	$t_{\mathrm{1}}\sim $ B(0.8, 1.2)	100	100	100	100	100	100
	***G*(2, 0.4)**	$t_{\mathrm{1}}\sim $ B(0.8, 1.2)	100	100	100	100	100	100
	***G*(2, 40)**	$t_{\mathrm{1}}\sim $ B(0.8, 1.2)	**100**	96	92	93	95	99
		$t_{\mathrm{3}}\sim $ B(0.525, 0.575)						
	$G$(2, 4)	$t_{\mathrm{1}}\sim $ B(0.8, 1.2)	**100**	94	85	86	91	98
		$t_{\mathrm{3}}\sim $ B(0.525, 0.575)						
	***G*(2, 0.4)**	$t_{\mathrm{1}}\sim $ B(0.8, 1.2)	**99**	93	83	84	90	97
		$t_{\mathrm{3}}\sim $ B(0.525, 0.575)						
	$G$(2, 4)	$t_{\mathrm{1}}\sim $ B(0.8, 1.2)	**82**	52	68	63	53	73
		***t*** $_{\mathrm{\mathbf{3}}}\sim $ **B(0.575, 0.625)**						
**AR**	$G$(2, 4)	$t_{\mathrm{1}}\sim $ B(0.8, 1.2)	**100**	**100**	0	0	**100**	**100**
	$G$(2, 4)	$t_{\mathrm{1}}\sim $ B(0.8, 1.2)	95	92	38	43	92	**98**
		$t_{\mathrm{3}}\sim $ B(0.525, 0.575)						

*Notes:*Coverage is averaged over the 100 replicates and over the 8 internal nodes on the tree. See caption of Table 1 for more details.

Use of an incorrect rate prior $\bar{\mu}\sim G$(2, 40), with the rate ~ 10 times too slow, and with a single calibration on the root led to seriously biased time estimates for all partition schemes (Fig. 2A). For example, the relative error with the slow-rate prior was 0.177 and 0.204 for partition schemes C and CP, in comparison with 0.028 and 0.046 with the correct rate prior (Table 1). Moreover, the use of the slow-rate prior produced misleadingly precise estimates (Tables 1 and 2), since the estimates are far from the true values and for many nodes the true ages were not within the HPD intervals for all partition schemes (Fig. 2A). For example, the age of the root (true age $=$ 100) was estimated at 114, 115, 116, 115, 117, and 115 for schemes C, CP, PF, G, GCP, and CS, respectively, but the HPD interval had a coverage probability of 66% for CS, 48% for scheme C (concatenation), and 0% for the other schemes (Table 4). In terms of the overall measure MSE, all partition schemes performed poorly with the mis-specified slow-rate prior when a single calibration is used on the root, with scheme G to be better than others (Table 1). The use of an additional correct calibration on node 3 improved the time estimates with the slow rate prior for all partition schemes. The fast-rate prior, $\bar{\mu}\sim G$(2, 0.4), with the rate to be ten times too fast, gave similar estimates to the correct rate prior, especially when two calibrations are used (Tables 1 and 2).

**Table 4.** Average coverage for nodes 1 (root) and 4 for different partitioning strategies when the clock is seriously violated

Model	$\bar{\mu}\sim$	Calibration	C (1P)	CP (2P)	G (50P)	GCP (100P)	PF (V)	CS (V)
IR	***G*(2, 40)**	$t_{\mathrm{1}}\sim $ B(0.8, 1.2)	48	0	0	0	0	**66**
			**73**	4	56	2	1	53
	$G$(2, 4)	$t_{\mathrm{1}}\sim $ B(0.8, 1.2)	100	100	100	100	100	100
			100	100	100	100	100	100
	***G*(2, 0.4)**	$t_{\mathrm{1}}\sim $ B(0.8, 1.2)	100	100	100	100	100	100
			100	100	100	100	100	100
	***G*(2, 40)**	$t_{\mathrm{1}}\sim $ B(0.8, 1.2)	**100**	93	99	96	89	**100**
		$t_{\mathrm{3}}\sim $ B(0.525, 0.575)	**100**	99	99	**100**	**100**	99
	$G$(2, 4)	$t_{\mathrm{1}}\sim $ B(0.8, 1.2)	**99**	82	**99**	80	68	92
		$t_{\mathrm{3}}\sim $ B(0.525, 0.575)	**99**	**99**	90	96	**99**	**99**
	***G*(2, 0.4)**	$t_{\mathrm{1}}\sim $ B(0.8, 1.2)	**99**	78	98	79	61	90
		$t_{\mathrm{3}}\sim $ B(0.525, 0.575)	**99**	**99**	84	94	**99**	**99**
	$G$(2, 4)	$t_{\mathrm{1}}\sim $ B(0.8, 1.2)	**92**	85	57	65	85	**92**
		***t*** $_{\mathrm{\mathbf{3}}}\sim$ **B(0.575, 0.625)**	**86**	22	78	58	30	65
**AR**	$G$(2, 4)	$t_{\mathrm{1}}\sim $ B(0.8, 1.2)	**100**	**100**	0	0	**100**	**100**
			**100**	**100**	0	0	99	**100**
	$G$(2, 4)	$t_{\mathrm{1}}\sim $ B(0.8, 1.2)	**100**	98	0	0	91	92
		$t_{\mathrm{3}}\sim $ B(0.525, 0.575)	96	97	64	74	**99**	**99**

*Notes:* Coverage for each node is averaged over the 100 replicates. The first row in each cell refers to node 1 and the second to node 4. See caption of Table 1 for more details.

We then explored time estimates in case of an incorrect calibration $0.575 < t_{3} < 0.625$ on node 3 (true age $t_{\mathrm{3}} = 0.55$), in addition to the correct calibration on thed root (Fig. 2C’). The accuracy of time estimates was worse for all partition schemes than when correct calibrations were used. For example, the relative error for schemes C and CP were 0.072 and 0.080, respectively, compared with 0.028 and 0.046 when a single calibration was used on the root, with the GCP scheme having the smallest relative error (Table 1). The precision of time estimates was higher than under a single calibration on the root for all partition schemes with the PF and GCP schemes achieving the highest precision (Table 1). In general, all node ages were overestimated for all partition schemes owing to the incorrect informative calibration on node 3. The age estimate of node 3 was most seriously affected, with the HPD interval failing to include the true age for all partition schemes. Overall, the GCP scheme had the highest accuracy and precision but the coverage probability is low (63%, Table 3).

We also analyzed the simulated data sets with the AR model (Fig. 2D and 2D’). In that case, the time estimates showed considerable differences among the partition schemes. With a single calibration on the root, increasing the number of partitions produced older and biased time estimates for all nodes (Fig. 2D). For example, the relative error for scheme C (concatenation) was 0.060 while it was ~ 7 times higher (0.435) for scheme G (Table 1). Moreover, the highly partitioned schemes (G and GCP) led to misleadingly high precision (Fig. 2D, Table 1). With the addition of a correct calibration on node 3, the accuracy of time estimates was improved, particularly for schemes G and GCP. However, the ages of the deep nodes (nodes 1 and 2) were more severely underestimated as the number of partitions increased while those of younger nodes were more severely overestimated (Fig. 2D’). This is probably because the calibration on node 3 was more informative (with uncertainty, defined as the 95% prior interval width divided by the mid-value, at 10%) than the one on the root (uncertainty 40%). Whatever the calibration strategy, a highly partitioned scheme (PF, G, or GCP) led to seriously biased time estimates (Fig. 2D & D’) with small coverage probabilities (Table 3). The mis-specified rate prior thus caused serious problems in divergence time estimation (cf. Fig. 2B’ vs. Fig. 2D’).

### Results from Simulation When the Clock Is Slightly Violated

The relative error, relative HPD width, and MSE averaged over the replicates and internal nodes for data simulated with slight clock violation ($\sigma^{\mathrm{2}} = 0.01$) are shown in Supplementary Table S1 available on Dryad, while the results for nodes 1 and 4 are shown in Supplementary Table S2 available on Dryad. Time estimates showed similar trends to those under serious clock violation but were more precise and accurate (Fig. 3), indicating that time estimation is easier when the clock roughly holds. For example, the relative error in case of a single calibration on the root with correct rate prior for partition scheme C (concatenation) was 0.019, compared with 0.028 with serious clock violation, and the relative HPD width was 0.43, in comparison with 0.50 (cf. Tables 5 and 1). In general, posterior time estimates were more similar among partition schemes than in the case of serious clock violation.

The effect of an incorrect rate prior was also the same as when the clock is seriously violated, with the slow-rate prior producing less accurate estimates than the correct rate prior, for all partition schemes. When two correct calibrations were used with the correct rate prior the time estimates were more precise than when a single calibration was used (Supplementary Table S1 available on Dryad). For example, the relative HPD width for nodes 1 and 4 with the C scheme was 0.16 and 0.13, respectively, with two correct calibrations, while they were 0.40 and 0.43 with a single calibration in the root (Supplementary Table S2 available on Dryad). When an incorrect calibration was used on node 3 all node ages were slightly overestimated for all partition schemes, as in the case of serious clock violation.

With the incorrect AR model the time estimates showed the same pattern as in the case of serious clock violation, although the differences among partition schemes were smaller. With a single calibration on the root time estimates under partition schemes C, CP, PF, G, and CS were close to the true values while time estimates under the GCP scheme were older and less accurate, especially for the deep nodes (Fig. 3D). Adding a correct calibration on node 3 improved time estimates for all partition schemes (Fig. 3D’). However, all except the C and CS schemes tended to give younger and less accurate estimates for the deep nodes.

Overall, with the slight clock violation the results show similar trends to those with serious clock violation, but the effects are much less serious. Use of the incorrect rate-drift model (AR vs. IR), incorrect rate prior (too high or too low rates), and incorrect fossil calibrations all had less damaging effects on posterior divergence time estimates when the clock is only slightly violated.

### Divergence Times of Plants

**Figure 4.**
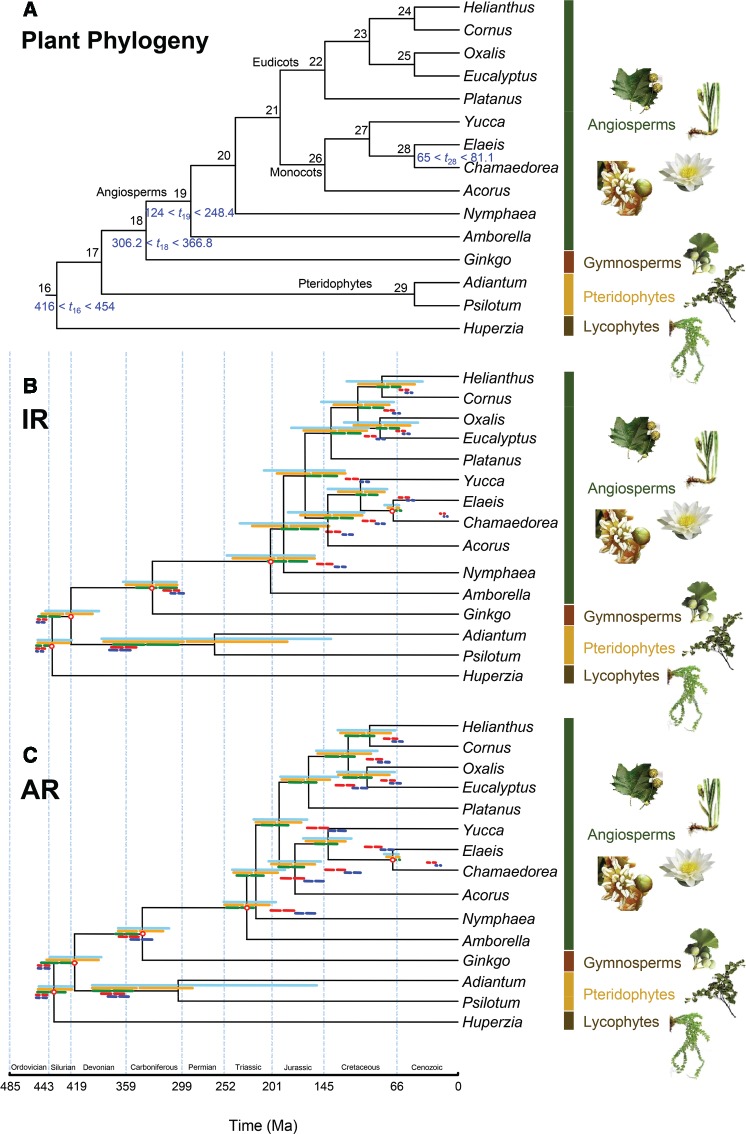
A) Phylogeny of 15 plant species, based on Magallon et al. (2013) and Ruhfel et al. (2014) with fossil calibrations. Nodes are numbered from 16 to 29. Fossil calibrations for five nodes are shown in blue next to the nodes. The fossil bounds are soft, with 5% probability for the true age to be outside the bounds (2.5% probability on each side). The calibrations are from Clarke et al. (2011) and Zanne et al. (2014). B) Posterior divergence times for the plant phylogeny using six partition schemes. The independent-rates model (IR) was used. Horizontal bars represent the 95% HPD intervals with gaps denoting the posterior mean. The partition schemes are (from top to bottom): (i) concatenation and ClockstaR (C and CS, 1 partition); (ii) codon positions (CP, 2 partitions); (iii) PartitionFinder (PF, 11 partitions); (iv) gene (G, 78 partitions); and (v) both gene and codon positions (GCP, 156 partitions). The timetree shown in black was estimated using scheme C (1 partition). Calibrated nodes are indicated by red circles. C) Same as B using the autocorrelated-rates model (AR).

We estimated the divergence times of fifteen plant species using the six partition schemes with the given tree topology of Figure 4A. The number of partitions is 1 for C and CS, 2 for CP, 11 for PF, 78 for G, and 156 for GCP. Note that ClockstaR inferred 1 partition while PartitionFinder suggested 11. The posterior means and 95% HPD intervals of divergence times are shown in Table 5. Time estimates were very similar for the three rate priors $G$(1, 100), $G$(1, 10) and $G$(1, 1), possibly because several calibrations were applied on a large phylogeny, so that the overall rate was well-constrained. Thus only the estimates under the prior $\bar{\mu}\sim G$(1, 10) are reported in Figure 4.

**Table 5.** Posterior means and 95% credibility intervals (in parentheses) of divergences times (myr) using different partition schemes for the plant data set

Node	Clade		Prior		CP (2P)	C and CS (1P)		PF (11P)			G (78P)	GCP (156P)	
Independent-rates model													
16	Root	437	(417, 455)	442	(421, 456)	440	(419, 456)	451	(440, 457)	453	(448, 457)	454	(450, 458)
17	*Helianthus*/*Psilotum*	412	(386, 443)	427	(397, 452)	420	(389, 448)	445	(431, 455)	452	(446, 456)	453	(449, 457)
18	Angiosperms/*Ginkgo*	337	(306, 367)	329	(304, 361)	332	(305, 363)	326	(305, 349)	310	(302, 319)	305	(297, 311)
19	Angiosperms	186	(124, 249)	197	(155, 245)	204	(155, 251)	184	(164, 206)	144	(136, 152)	127	(122, 133)
20	*Helianthus*/*Nymphaea*	172	(111, 237)	183	(40, 223)	189	(142, 237)	172	(152, 191)	135	(128, 143)	120	(114, 125)
21	*Helianthus*/*Acorus*	158	(102, 223)	159	(122, 197)	166	(123, 211)	147	(131, 164)	115	(109, 121)	101	(97, 106)
22	Eudicots	134	(76, 211)	131	(99, 167)	138	(97, 180)	121	(106, 137)	95	(89, 101)	84	(80, 88)
23	*Helianthus*/*Eucalyptus*	111	(38, 180)	103	(74, 134)	109	(70, 148)	93.1	(81, 107)	75	(70, 80)	67	(63, 70)
24	*Helianthus*/*Cornus*	71	(0, 132)	79	(47, 108)	82	(39, 120)	74	(63, 87)	59	(54, 63)	53	(50, 57)
25	*Oxalis*/*Eucalyptus*	71	(0, 131)	81	(52, 113)	85	(44, 123)	76	(64, 88)	63	(58, 67)	57	(53, 60)
26	Monocots	130	(78, 192)	137	(103, 171)	141	(102, 184)	129	(114, 144)	96	(90, 103)	84	(80, 89)
27	*Yucca*/*Chamaedorea*	101	(66, 152)	103	(80, 130)	106	(77, 141)	98	(88, 110)	59	(53, 64)	51	(48, 56)
28	*Elaeis*/*Chamaedorea*	74	(65, 81)	68	(64, 76)	70	(64, 80)	65	(62, 67)	17	(15, 20)	13	(12, 15)
29	Ferns	146	(0, 369)	296	(185, 385)	264	(138, 386)	339	(303, 374)	362	(349, 376)	368	(356, 378)
Autocorrelated-rates model													
16	Root	437	(417, 455)	439	(418, 455)	438	(418, 455)	443	(426, 456)	452	(446, 457)	453	(446, 458)
17	*Helianthus*/*Psilotum*	412	(386, 443)	419	(390, 446)	416	(387, 443)	433	(414, 452)	450	(443, 455)	450	(443, 455)
18	Angiosperms/*Ginkgo*	337	(306, 367)	347	(320, 368)	342	(313, 368)	361	(348, 371)	358	(347, 367)	344	(332, 355)
19	Angiosperms	186	(124, 249)	232	(204, 253)	229	(197, 254)	237	(222, 251)	191	(178, 203)	166	(155, 176)
20	*Helianthus*/*Nymphaea*	172	(111, 237)	221	(195, 243)	219	(187, 245)	226	(210, 240)	179	(167, 192)	156	(146, 166)
21	*Helianthus*/*Acorus*	158	(102, 223)	196	(170, 219)	162	(129, 192)	199	(184, 213)	152	(141, 164)	131	(122, 140)
22	Eudicots	134	(76, 211)	165	(139, 191)	119	(87, 154)	168	(153, 183)	122	(113, 132)	106	(99, 114)
23	*Helianthus*/*Eucalyptus*	111	(38, 180)	122	(94, 152)	95	(68, 130)	127	(112, 143)	90	(82, 98)	80	(74, 87)
24	*Helianthus*/*Cornus*	71	(0, 132)	100	(73, 127)	99	(69, 131)	106	(91, 121)	73	(66, 80)	66	(60, 71)
25	*Oxalis*/*Eucalyptus*	71	(0, 131)	102	(74, 129)	177	(149, 205)	108	(93, 124)	76	(69, 823)	68	(63, 74)
26	Monocots	130	(78, 192)	179	(155, 202)	141	(116, 167)	181	(167, 194)	133	(122, 143)	114	(105, 122)
27	*Yucca*/*Chamaedorea*	101	(66, 152)	142	(122, 165)	71	(64, 80)	143	(131, 155)	92	(83, 100)	77	(70, 84)
28	*Elaeis*/*Chamaedorea*	74	(65, 81)	69	(64, 77)	303	(153, 396)	65	(63, 68)	29	(24, 33)	21	(18, 24)
29	Ferns	146	(0, 369)	347	(288, 397)	162	(129, 192)	375	(353, 396)	375	(362, 387)	369	(357, 380)

*Notes:*Node numbers are according to Figure 4. The rate prior was $\bar{\mu} \sim G$(1, 10).

Figure 4B and C shows the posterior means and 95% HPD intervals of divergence times estimated under the six partition schemes. The differences in time estimates among the partition schemes were very large, even for some nodes with calibration. As in the analysis of the simulated data with serious clock violation, fine partitioning (schemes G and GCP) led to very narrow posterior intervals and high precision. Under the IR model, the estimated ages of the deep nodes (i.e., nodes 16, 17, and 29) became older as the number of partitions increased, whereas those of the other nodes became younger. For example, the age of pteridophytes (node 29) varied between 264 Ma (C and CS schemes) and 368 Ma (GCP scheme) while the age of angiosperms (node 19) varied between 127 Ma (GCP scheme) and 204 Ma (C and CS schemes) (Table 5). The time estimates for the angiosperms were within the calibration bounds with the youngest estimate to be very close to the minimum bound (124 Ma). However, for node 28 the posterior time estimates varied from 13 Ma (GCP scheme) to 70 Ma (C and CS schemes) with the estimates under the G and GCP schemes to be well below the minimum bound (65 Ma).

The estimates under the AR model showed similarly large discrepancies among partition schemes. For example, the posterior age estimates of the root varied from 438 Ma (C and CS schemes) to 453 Ma (GCP scheme) and the age of node 29 from 303 Ma (schemes C and CS) to 375 Ma (schemes PF and G). The age estimates of the deepest nodes became older as the number of partitions increased. The time estimates under the AR model were in general older than those under the IR model. For example, the posterior mean of node 29 was 264 Ma and 296 Ma with the schemes C, CS, and CP, respectively, under the IR model, compared with 303 Ma and 347 Ma, under the AR model (Table 5).

In general, the differences among the partition schemes were large, similar to the analysis of simulated data with serious clock violation under the incorrect rate-drift model. The highly partitioned schemes G and GCP tended to produce precise estimates, far from those for the other three schemes. In some cases those estimates were outside the calibration bounds (e.g., node 28) irrespective of the clock model, raising concerns about their accuracy. Note that the posterior estimate for the rate drift parameter $\sigma^{\mathrm{2}}$ using the C scheme and the IR model was 0.58, much larger than the value we used in the simulation (0.25), indicating the plant sequence data show much more serious clock violation than in the simulated data.

## Discussion

### When the Molecular Clock Is Seriously Violated, Partition Schemes Have a Major Impact on Divergence Time Estimation

Previously, Poux et al. (2008) and Voloch and Schrago (2012) examined the impact of partition schemes on divergence time estimation and found little difference in time estimates among partition schemes. We suggest that this is because those studies used many calibrations and focused on the analyses of closely related species, for which the molecular clock approximately holds. Indeed the infinite-sites theory for divergence time estimation under the molecular clock (Yang and Rannala 2006; dos Reis and Yang 2013) predicts that the choice of partition schemes is unimportant when the clock roughly holds. If the strict clock holds, the branch lengths will be proportional among partitions, and either one or many partitions will provide about the same amount of information concerning the relative node ages (if each partition has a substantial number of sites), and partition schemes should have little effect on posterior time estimation (Yang and Rannala 2006; dos Reis and Yang 2013).

However, if the clock is seriously violated and a relaxed-clock model is assumed, partition schemes become very important. According to the infinite-sites theory, with serious clock violation the use of many partitions is essential for improving the precision of posterior time estimates (Rannala and Yang 2007; Zhu et al. 2015). The different partitions act like replications of the rate-drift process, providing essential information to tease apart the effects of divergence times and local rate variation. For example, a long branch in a particular gene tree is compatible with both a long time duration and a high rate, but a high rate and a short time duration is more likely if the same branch is short in other partitions. Different partition schemes and the number of partitions are then expected to have a major impact on divergence time estimation when the clock is seriously violated. Results from our simulation and real-data analyses suggest that coarse partition schemes (e.g., C, CP schemes) produce uncertain time estimates with wide-HPD intervals but achieve high-coverage probabilities. In contrast, fine partitions (e.g., PF, G, GCP schemes) produced narrow posterior intervals with high precision (Table 1). When the calibrations and rate priors are correct, the finest partition scheme GCP gives overall the best performance (Table 1). Note that our simulation generates substitution rate variation among codon positions and among sites of the same codon positions, and independent rate drift among partitions. While a nucleotide-based model may not fit a codon model perfectly, we expect the G partition scheme (50 partitions for 50 genes) should be a good approximation, as the HKY$+\Gamma $ model can deal with the rate variation among sites of the same gene. We note that this G scheme as well as the GCP scheme worked fairly well when the rate-drift model is correct and the fossil calibrations are correct. However, if the calibrations are incorrect or the prior is mis-specified, fine partitioning still produce narrow intervals, but the narrow intervals may fail to include the true ages as the time estimates may be seriously biased. Incorrect calibrations, in particular, exert a significant impact on time estimates. If a highly partitioned scheme is used in combination with incorrect prior assumptions about the relative rates or incorrect fossil calibrations, posterior time estimates may be highly precise and seriously biased, although the direction of the bias depends on the locations and precision of fossil calibrations on the tree (Fig. 2D and 2D’).

### Automated Partitioning (PartitionFinder and ClockstaR) Did Not Produce Consistently Superior Time Estimates

In our simulation, the two automated approaches to selecting the best-fitting partition scheme, PartitionFinder and ClockstaR, did not appear to outperform simple schemes of partitioning by gene or codon position. PartitionFinder tests for the goodness of fit of the substitution and partition models while in relaxed-clock dating analyses, the most important factors may be those that affect the estimation of branch lengths in gene trees for the partitions such as the relative rates for partitions and the different patterns of rate drift among partitions. Note that factors that affect model adequacy or the goodness of fit of a model (as judged by the likelihood values or information criteria) may be very different from those that affect model robustness or sensitivity of posterior estimates. For example, accommodating the transition/transversion rate ratio or the different frequencies of the four nucleotides is known to improve the fit of the model hugely, but they do not impact the estimation of branch lengths as much as among-site rate variation (Yang et al. 1994). In relaxed clock dating, the assumptions about how the evolutionary rate drifts over branches in the different partitions should be important. In this regard, the linking option of PartitionFinder that we used (which implies that branch lengths are proportional across partitions) may not be very appropriate, and it is unclear whether unlinking branch lengths may cause PartitionFinder to infer more partitions or lead to better performance. In our simulation, the branch rates were independently generated for the 50 protein-coding genes, so that the use of 50 partitions may be justifiable. However, a major consideration of our simulation is to allow for mis-specified rate-drift models and incorrect fossil calibrations, as such mis-specifications appear to be commonplace in modern dating analysis. In this regard, we note that none of the biologically motivated partition schemes (CP, G, and GCP) achieved consistently better performance than the other schemes, and in particular the G scheme tended to produce posterior intervals that are too narrow and fail to include the true ages.

ClockstaR attempts to determine the right number of “molecular clocks” for the given data set, by grouping data blocks with proportional branch lengths into the same partition (Duchêne et al. 2014). Duchêne and Ho (2014) simulated sequence data with 1, 2, or 3 true partitions and found that ClockstaR was most often able to select the correct number of partitions and to produce much better time estimates than random partitioning. Our simulation in this study is more complex and involves far more potential partitions. Furthermore, partition schemes CP, G, and GCP, which we used for comparison, are based on genes and/or codon positions and should be superior to random partitioning. We found the inference by ClockstaR of only one partition for the simulated data sets with serious clock violation (Supplementary Fig. S1 available on Dryad) and for the empirical plant data set to be surprising. As the rates for branches were independently generated for the 50 protein-coding genes in the simulated data sets, the use of 50 partitions may be justifiable, and the use of one partition may lead to underfitting. Similarly in the analysis of data for 24 pinniped species by Duchêne and Ho (2014), ClockstaR grouped 15 nuclear genes into one partition and 13 mitochondrial genes into another. The precise reasons for this behavior of ClockstaR are not well understood, but we note that the distance metric used in the method is based on the sum of squared differences between (scaled) branch lengths (Duchêne et al. 2014, Equation 1). This metric ignores the variances of branch length estimates and is dominated by large branch lengths. In contrast, Bayesian clock dating analysis naturally accommodates such differences in variance in calculation of the likelihood function, and extremely short branches may be as problematic as extremely long branches in causing serious violations of the clock and in affecting posterior time estimation. It may be interesting to examine whether a weighted distance metric, using the reciprocal of the variance of the branch length as the weight, may lead to improved performance.

### Bias, Variance, Concatenation, and Partitioning in Relaxed Clock Dating Analysis

We note that the effects of concatenation versus partitioning are opposite in phylogenetic tree reconstruction and in divergence time estimation. In phylogeny estimation, concatenation typically leads to high-support values (such as high-bootstrap proportions or posterior probabilities) for inferred clades, even if the clades may be spurious. This is particularly the case for species tree estimation when the different genes undergo incomplete lineage sorting due to ancestral polymorphism (Edwards et al. 2016; Xu and Yang 2016). The problem with concatenation lies in its use of an underspecified model that fails to account for the heterogeneity in the substitution process and in the gene tree topology and branch lengths among the genes or partitions, leading to biased and overconfident species tree estimates. The pattern is similar to bias-variance trade-off in which use of a simplistic underparametrized model leads to smaller variance and larger bias. In contrast, in estimation of divergence times under relaxed-clock models, concatenation is seen to produce wider posterior intervals, with higher coverage but lower precision, than partition analysis. This pattern may be understood through the infinite-sites theory (Yang and Rannala 2006; Rannala and Yang 2007; dos Reis and Yang 2013; Zhu et al. 2015). Molecular clock dating analysis involves a serious confounding effect between times and rates. When the clock holds, even one gene or partition (with many sites) can be very informative about the relative node ages. Increasing the number of sites in one gene will be sufficient for the posterior time estimates to converge to the infinite-data limit (in which the posterior becomes one-dimensional), whereas using more genes or partitions adds little extra information, since the rates (and thus the branch lengths) are proportional among partitions (Yang and Rannala 2006). However, when the clock is violated and a relaxed-clock model is assumed, the relative node ages are confounded with the local branch rates for each gene or partition. Then different partitions act as independent realizations of the rate-drift process, providing the information to resolve the confounding effect of the relative node ages and the local branch rates. Thus adding loci tends to be much more effective than adding sites for each locus in improving the precision of posterior time estimates under relaxed-clock models (Zhu et al. 2015). The main problem of concatenation is then its underuse of the information in the multi-partition data concerning the rate-drift process and the divergence times.

We note that evolutionary rate-drift models implemented in current computer programs all ignore lineage effects (species or genome effects) that affect the rates of all genes in the whole genome. Such effects may be due to life history characteristics such as generation times, population sizes, and so forth that may affect the evolutionary rate throughout the genome. Our simulation similarly ignores such lineage effects, as the rate drift process assumed in the simulation is independent among genes or partitions. Lineage effects would create strong correlations in evolutionary rates among genes or partitions. It appears to be straightforward to implement rate-drift models with lineage effects, and simulation under such models is simple as well. Nevertheless genome-wide rate changes will be confounded with the prior model on divergence times on the species tree, thus creating a dire situation of model unidentifiability, and increasing the number of partitions will then not improve the precision of posterior time estimates. See dos Reis et al. (2016) and Donoghue and Yang (2016) for further discussions of the shortcomings of current rate-drift models.

### Limitations of Our Simulation Strategy

Protein-coding genes are commonly used in molecular clock dating analysis (Meusemann et al. 2010; dos Reis et al. 2012; Misof et al. 2014). We thus simulated gene alignments under a codon model that allows for different $\omega $ ratios across codons. Because of selection against nonsynonymous changes, the model is expected to introduce rate variation among codon positions and among sites of the same gene. We did not simulate changes to selective pressure or to the efficacy of selection on particular lineages, as accounted for by the branch-site model (Yang and Nielsen 2002). We also assumed equal codon frequencies for all genes in our simulation. This is unrealistic but may not have a major effect on the estimation of the branch lengths and of divergence times. We assumed the same gene tree topology for all genes and ignored factors that may cause the gene trees to differ from the species tree, such as ancestral coalescent processes that cause gene tree-species tree conflicts (Nichols 2001). Designing partition strategies to deal with both gene tree-species tree conflicts and the heterogeneity in the substitution process will be challenging. Finally, as mentioned earlier, our simulation assumed independent rate drift among partitions and ignored lineage effects, which are expected to have large impact on divergence time estimation.

## Supplementary Material

Data available from the Dryad Digital Repository: http://dx.doi.org/10.5061/dryad.d7839.
